# Supplementary material for: Hot spot prediction in protein-protein interactions by an ensemble system
Source: BMC Syst Biol. 2018 Dec 31;12(Suppl 9):132. doi: 10.1186/s12918-018-0665-8 (PMC6311905; doi:10.1186/s12918-018-0665-8)
Supplement: Supplementary file 2 — All relevant MATLAB code. A simple Matlab implement of our predictor. (ZIP 19,742 kb) [file 12918_2018_665_MOESM2_ESM.zip › Additional file 2/Read ASA/ASA website/dbMPIKT/1NVUR.html]

 NetSurfP - Protein Surface Accessibility and Secondary Structure Predictions

  

|  |  |  |
| --- | --- | --- |
|  |  | NetSurfP - Protein Surface Accessibility and Secondary Structure Predictions  Technical University of Denmark |

  


---

```
# For publication of results, please cite:
# A generic method for assignment of reliability scores applied to solvent accessibility predictions.
# Bent Petersen, Thomas Nordahl Petersen, Pernille Andersen, Morten Nielsen and Claus Lundegaard
# BMC Structural Biology 2009, 9:51 doi:10.1186/1472-6807-9-51
#
# Column 1: Class assignment - B for buried or E for Exposed - Threshold: 25% exposure, but not based on RSA
# Column 2: Amino acid
# Column 3: Sequence name
# Column 4: Amino acid number
# Column 5: Relative Surface Accessibility - RSA
# Column 6: Absolute Surface Accessibility
# Column 7: Z-fit score for RSA prediction
# Column 8: Probability for Alpha-Helix
# Column 9: Probability for Beta-strand
# Column 10: Probability for Coil
E M  1NVUR                  1    0.608 121.661  -0.820   0.003   0.003   0.994
E T  1NVUR                  2    0.458  63.511   1.267   0.004   0.085   0.910
E E  1NVUR                  3    0.457  79.785   1.921   0.004   0.514   0.481
B Y  1NVUR                  4    0.077  16.391   1.043   0.002   0.816   0.182
B K  1NVUR                  5    0.176  36.203   1.509   0.001   0.900   0.099
B L  1NVUR                  6    0.031   5.676   1.076   0.000   0.983   0.017
B V  1NVUR                  7    0.024   3.673   1.351   0.000   0.983   0.017
B V  1NVUR                  8    0.019   2.951   1.382   0.001   0.959   0.040
B V  1NVUR                  9    0.026   4.073   0.853   0.001   0.900   0.099
B G  1NVUR                 10    0.043   3.416  -0.468   0.004   0.420   0.576
B A  1NVUR                 11    0.197  21.687  -0.586   0.005   0.045   0.951
B G  1NVUR                 12    0.268  21.076  -1.424   0.018   0.019   0.964
B G  1NVUR                 13    0.217  17.046  -1.289   0.115   0.016   0.868
B V  1NVUR                 14    0.052   7.992   0.036   0.339   0.016   0.645
B G  1NVUR                 15    0.041   3.242  -1.428   0.430   0.016   0.555
B K  1NVUR                 16    0.090  18.492  -0.211   0.802   0.014   0.185
B S  1NVUR                 17    0.052   6.118  -0.091   0.879   0.010   0.111
B A  1NVUR                 18    0.034   3.736  -0.314   0.938   0.007   0.055
B L  1NVUR                 19    0.030   5.585   0.378   0.975   0.003   0.022
B T  1NVUR                 20    0.022   3.093   0.596   0.975   0.003   0.022
B I  1NVUR                 21    0.115  21.220   0.534   0.938   0.007   0.055
B Q  1NVUR                 22    0.107  19.074   1.130   0.938   0.007   0.055
B L  1NVUR                 23    0.065  11.828   0.019   0.938   0.007   0.055
B I  1NVUR                 24    0.063  11.729   0.332   0.802   0.014   0.185
E Q  1NVUR                 25    0.506  90.372   0.894   0.600   0.003   0.397
E N  1NVUR                 26    0.587  85.937  -0.136   0.176   0.004   0.820
E H  1NVUR                 27    0.457  83.128   1.317   0.113   0.043   0.844
B F  1NVUR                 28    0.062  12.484   0.006   0.052   0.084   0.864
E V  1NVUR                 29    0.388  59.697   1.632   0.053   0.043   0.903
E D  1NVUR                 30    0.705 101.619   0.081   0.053   0.043   0.903
E E  1NVUR                 31    0.645 112.751  -0.054   0.053   0.043   0.903
B Y  1NVUR                 32    0.224  47.847   0.598   0.052   0.084   0.864
E D  1NVUR                 33    0.533  76.791   0.788   0.052   0.084   0.864
B P  1NVUR                 34    0.157  22.207   0.043   0.052   0.084   0.864
B T  1NVUR                 35    0.145  20.111   0.008   0.069   0.386   0.545
B I  1NVUR                 36    0.124  22.940   0.744   0.079   0.592   0.329
B E  1NVUR                 37    0.172  30.013  -0.686   0.079   0.592   0.329
B D  1NVUR                 38    0.140  20.203  -0.051   0.023   0.655   0.322
B S  1NVUR                 39    0.271  31.796  -0.572   0.018   0.846   0.136
B Y  1NVUR                 40    0.093  19.853   0.507   0.001   0.900   0.099
E R  1NVUR                 41    0.362  82.852   1.328   0.001   0.959   0.040
E K  1NVUR                 42    0.243  49.985   1.602   0.001   0.959   0.040
E Q  1NVUR                 43    0.403  71.887   1.606   0.000   0.983   0.017
B V  1NVUR                 44    0.065  10.037   1.361   0.000   0.983   0.017
E V  1NVUR                 45    0.492  75.590   0.743   0.000   0.983   0.017
B I  1NVUR                 46    0.107  19.758   0.807   0.001   0.959   0.040
E D  1NVUR                 47    0.682  98.291   0.742   0.004   0.138   0.858
E G  1NVUR                 48    0.799  62.905   0.316   0.003   0.003   0.994
E E  1NVUR                 49    0.535  93.552   1.425   0.003   0.718   0.279
E T  1NVUR                 50    0.455  63.067   2.001   0.001   0.959   0.040
B C  1NVUR                 51    0.044   6.234   1.336   0.006   0.962   0.032
E L  1NVUR                 52    0.295  54.106   2.485   0.006   0.962   0.032
B L  1NVUR                 53    0.026   4.687   1.021   0.001   0.959   0.040
B D  1NVUR                 54    0.114  16.485   1.146   0.011   0.918   0.071
B I  1NVUR                 55    0.026   4.828   0.866   0.011   0.918   0.071
B L  1NVUR                 56    0.087  15.966   0.982   0.018   0.846   0.136
B D  1NVUR                 57    0.086  12.421   0.286   0.022   0.552   0.426
B T  1NVUR                 58    0.124  17.185   0.252   0.056   0.142   0.802
B G  1NVUR                 59    0.148  11.624  -0.636   0.115   0.016   0.868
E G  1NVUR                 60    0.271  21.304  -0.743   0.257   0.016   0.727
B Q  1NVUR                 61    0.239  42.632   0.340   0.522   0.016   0.462
E E  1NVUR                 62    0.369  64.447  -0.268   0.622   0.015   0.363
E E  1NVUR                 63    0.370  64.691   0.624   0.660   0.049   0.291
B Y  1NVUR                 64    0.087  18.699   0.011   0.660   0.049   0.291
E S  1NVUR                 65    0.399  46.716   1.157   0.660   0.049   0.291
B A  1NVUR                 66    0.208  22.933  -1.032   0.522   0.016   0.462
B M  1NVUR                 67    0.103  20.710   0.116   0.522   0.016   0.462
B R  1NVUR                 68    0.173  39.548   0.568   0.522   0.016   0.462
E D  1NVUR                 69    0.486  70.047  -0.247   0.522   0.016   0.462
B Q  1NVUR                 70    0.226  40.328  -0.181   0.561   0.047   0.393
B Y  1NVUR                 71    0.154  32.867  -0.355   0.502   0.102   0.396
B M  1NVUR                 72    0.097  19.390   0.374   0.307   0.165   0.527
E R  1NVUR                 73    0.287  65.723   0.252   0.199   0.152   0.649
E T  1NVUR                 74    0.337  46.742  -0.771   0.053   0.043   0.903
B G  1NVUR                 75    0.036   2.865  -0.731   0.018   0.088   0.893
B E  1NVUR                 76    0.175  30.520   0.138   0.005   0.336   0.660
B G  1NVUR                 77    0.022   1.763   0.663   0.002   0.816   0.182
B F  1NVUR                 78    0.018   3.693   1.362   0.001   0.959   0.040
B L  1NVUR                 79    0.018   3.277   1.587   0.001   0.959   0.040
B C  1NVUR                 80    0.018   2.569   1.741   0.000   0.983   0.017
B V  1NVUR                 81    0.023   3.489   1.135   0.001   0.959   0.040
B F  1NVUR                 82    0.060  12.142   0.999   0.001   0.900   0.099
B A  1NVUR                 83    0.071   7.857  -0.579   0.002   0.816   0.182
B I  1NVUR                 84    0.062  11.414   0.540   0.005   0.336   0.660
E N  1NVUR                 85    0.292  42.734   0.151   0.005   0.045   0.951
E N  1NVUR                 86    0.361  52.821   0.910   0.018   0.019   0.964
E T  1NVUR                 87    0.441  61.222   1.601   0.694   0.003   0.303
E K  1NVUR                 88    0.590 121.425   1.363   0.600   0.003   0.397
B S  1NVUR                 89    0.060   7.009   0.187   0.694   0.003   0.303
B F  1NVUR                 90    0.063  12.724  -0.121   0.923   0.002   0.076
E E  1NVUR                 91    0.585 102.252   1.546   0.970   0.001   0.030
E D  1NVUR                 92    0.369  53.231   1.500   0.970   0.001   0.030
B I  1NVUR                 93    0.017   3.071   1.318   0.970   0.001   0.030
E H  1NVUR                 94    0.459  83.419   1.605   0.988   0.000   0.012
E Q  1NVUR                 95    0.485  86.621   2.243   0.970   0.001   0.030
B Y  1NVUR                 96    0.040   8.548   0.557   0.988   0.000   0.012
B R  1NVUR                 97    0.113  25.991   1.463   0.988   0.000   0.012
E E  1NVUR                 98    0.584 102.077   2.061   0.988   0.000   0.012
B Q  1NVUR                 99    0.212  37.810   1.470   0.988   0.000   0.012
B I  1NVUR                100    0.014   2.553   1.552   0.970   0.001   0.030
E K  1NVUR                101    0.374  76.932   1.767   0.975   0.003   0.022
E R  1NVUR                102    0.683 156.384   1.552   0.938   0.007   0.055
B V  1NVUR                103    0.222  34.121   0.658   0.782   0.003   0.216
B K  1NVUR                104    0.273  56.177   0.027   0.321   0.003   0.675
E D  1NVUR                105    0.666  95.985  -0.593   0.053   0.005   0.942
E S  1NVUR                106    0.384  45.005  -0.308   0.016   0.005   0.979
E D  1NVUR                107    0.756 108.940  -0.447   0.016   0.005   0.979
E D  1NVUR                108    0.454  65.378   0.978   0.005   0.015   0.979
B V  1NVUR                109    0.028   4.365   1.096   0.004   0.197   0.799
B P  1NVUR                110    0.078  10.997   1.019   0.002   0.816   0.182
B M  1NVUR                111    0.033   6.663   0.931   0.001   0.959   0.040
B V  1NVUR                112    0.014   2.213   1.771   0.000   0.983   0.017
B L  1NVUR                113    0.014   2.600   1.739   0.001   0.959   0.040
B V  1NVUR                114    0.015   2.275   1.452   0.001   0.900   0.099
B G  1NVUR                115    0.023   1.779   0.077   0.004   0.616   0.381
B N  1NVUR                116    0.076  11.126  -0.586   0.022   0.359   0.619
B K  1NVUR                117    0.175  36.018   0.241   0.019   0.141   0.840
B C  1NVUR                118    0.165  23.124  -0.127   0.018   0.047   0.935
B D  1NVUR                119    0.226  32.509   0.021   0.018   0.047   0.935
B L  1NVUR                120    0.228  41.674   0.244   0.058   0.017   0.925
E A  1NVUR                121    0.672  74.087   0.717   0.058   0.017   0.925
E A  1NVUR                122    0.697  76.798   0.684   0.058   0.017   0.925
E R  1NVUR                123    0.273  62.403   1.068   0.053   0.043   0.903
E T  1NVUR                124    0.570  79.087   1.115   0.052   0.084   0.864
B V  1NVUR                125    0.059   9.114   0.768   0.018   0.088   0.893
E E  1NVUR                126    0.413  72.081   1.558   0.018   0.047   0.935
E S  1NVUR                127    0.489  57.264   1.241   0.923   0.002   0.076
E R  1NVUR                128    0.689 157.689   1.253   0.970   0.001   0.030
E Q  1NVUR                129    0.358  63.903   1.724   0.970   0.001   0.030
B A  1NVUR                130    0.028   3.130  -0.233   0.970   0.001   0.030
E Q  1NVUR                131    0.379  67.689   2.174   0.970   0.001   0.030
E D  1NVUR                132    0.575  82.829   1.970   0.970   0.001   0.030
B L  1NVUR                133    0.109  19.866   1.220   0.970   0.001   0.030
B A  1NVUR                134    0.018   2.006   0.129   0.970   0.001   0.030
E R  1NVUR                135    0.715 163.689   1.543   0.970   0.001   0.030
E S  1NVUR                136    0.643  75.324   1.394   0.858   0.002   0.139
E Y  1NVUR                137    0.262  56.011   1.517   0.406   0.004   0.590
E G  1NVUR                138    0.779  61.284   0.692   0.003   0.003   0.994
B I  1NVUR                139    0.048   8.824   0.501   0.005   0.015   0.979
E P  1NVUR                140    0.311  44.060   1.692   0.005   0.336   0.660
B Y  1NVUR                141    0.073  15.579   0.437   0.001   0.900   0.099
B I  1NVUR                142    0.078  14.393   0.503   0.011   0.918   0.071
B E  1NVUR                143    0.064  11.233   0.202   0.011   0.918   0.071
B T  1NVUR                144    0.030   4.203   0.211   0.021   0.756   0.223
B S  1NVUR                145    0.067   7.817  -0.008   0.021   0.451   0.528
B A  1NVUR                146    0.043   4.783  -1.279   0.125   0.227   0.648
B K  1NVUR                147    0.243  50.067   0.390   0.113   0.043   0.844
E T  1NVUR                148    0.472  65.536   0.335   0.058   0.017   0.925
E R  1NVUR                149    0.525 120.294  -0.627   0.109   0.005   0.886
E Q  1NVUR                150    0.445  79.441   1.261   0.321   0.003   0.675
B G  1NVUR                151    0.238  18.754   0.954   0.181   0.016   0.803
B V  1NVUR                152    0.019   2.936   1.528   0.923   0.002   0.076
E E  1NVUR                153    0.390  68.150   2.213   0.970   0.001   0.030
E D  1NVUR                154    0.436  62.756   2.065   0.970   0.001   0.030
B A  1NVUR                155    0.030   3.339   1.212   0.988   0.000   0.012
B F  1NVUR                156    0.022   4.375   1.301   0.988   0.000   0.012
E Y  1NVUR                157    0.323  69.004   2.216   0.970   0.001   0.030
E T  1NVUR                158    0.336  46.631   2.227   0.970   0.001   0.030
B L  1NVUR                159    0.029   5.383   1.061   0.988   0.000   0.012
B V  1NVUR                160    0.030   4.688   0.293   0.988   0.000   0.012
E R  1NVUR                161    0.432  98.928   2.108   0.988   0.000   0.012
E E  1NVUR                162    0.269  46.907   1.889   0.970   0.001   0.030
B I  1NVUR                163    0.028   5.254   0.186   0.923   0.002   0.076
E R  1NVUR                164    0.354  80.997   1.586   0.858   0.002   0.139
E Q  1NVUR                165    0.751 134.146   1.652   0.694   0.003   0.303
E H  1NVUR                166    0.713 129.749   0.509   0.016   0.005   0.979

Explain the output.  Go back.
```

---
